# Preneurosurgical Care for Patients With Trigeminal Neuralgia

**DOI:** 10.1001/jamanetworkopen.2025.58967

**Published:** 2026-02-12

**Authors:** Melanie Alfonzo Horowitz, Yuanxuan Xia, Salodin Al-Achkar, Michael Mugerwa, Megan Parker, Saket Myneni, Michael Lim, Christopher Jackson, Judy Huang, Risheng Xu, Chetan Bettegowda

**Affiliations:** 1 Department of Neurosurgery, Johns Hopkins University School of Medicine, Baltimore, Maryland; 2 Department of Neurosurgery, Stanford School of Medicine, Palo Alto, California

## Abstract

This cross-sectional study characterizes the care pathways of US patients with trigeminal neuralgia from the onset of their pain until their first neurosurgical consultation in terms of symptom duration.

## Introduction

Trigeminal neuralgia (TN) is characterized by attacks of severe sharp facial pain which may be difficult to distinguish from odontogenic pain.^1^ The similarities in pain character, combined with the lower prevalence of TN, may delay accurate diagnosis and referral to specialists. In this study, we sought to prospectively characterize the care pathways that US patients with TN follow from the onset of their pain until their first neurosurgical consultation and to examine associations with symptom duration.

## Methods

All adult patients (aged ≥18 years) seen for facial pain consistent with TN at a TN surgery center within a major academic hospital from March 1, 2024, to February 28, 2025, were prospectively surveyed regarding prior health care encounters for facial pain. Medical records supplemented survey data. Linear regression identified variables associated with increased symptom duration. Analyses were conducted in RStudio version 3.3.2 (R Foundation for Statistical Computing). The Johns Hopkins institutional review board approved the study with a waiver of informed consent because the study involved no more than minimal risk. The study followed the STROBE reporting guideline for cross-sectional studies. Statistical significance was set at 2-sided *P* ≤ .05.

## Results

The Figure depicts unique patient pathways through dentists and nondental practitioners prior to neurosurgical consultation. Among 197 patients (median age, 62.4 [IQR, 47.9-69.9] years; 127 [64.1%] female), 105 [53.3%] consulted a dentist and 31 [29.5%] of these underwent dental procedures for facial pain before index neurosurgical visit. Median time from dental appointment to neurosurgical consultation was 36 (IQR, 12-83) months. Of 105 dentists, 12 [11.4%] diagnosed TN, 27 [25.7%] referred patients to neurology, and 20 [18.9%] ruled out dental causes after completing workup. A prior dental appointment for TN pain, dental procedures, and dental diagnosis were not associated with increased symptom duration (Table).

**Figure.**
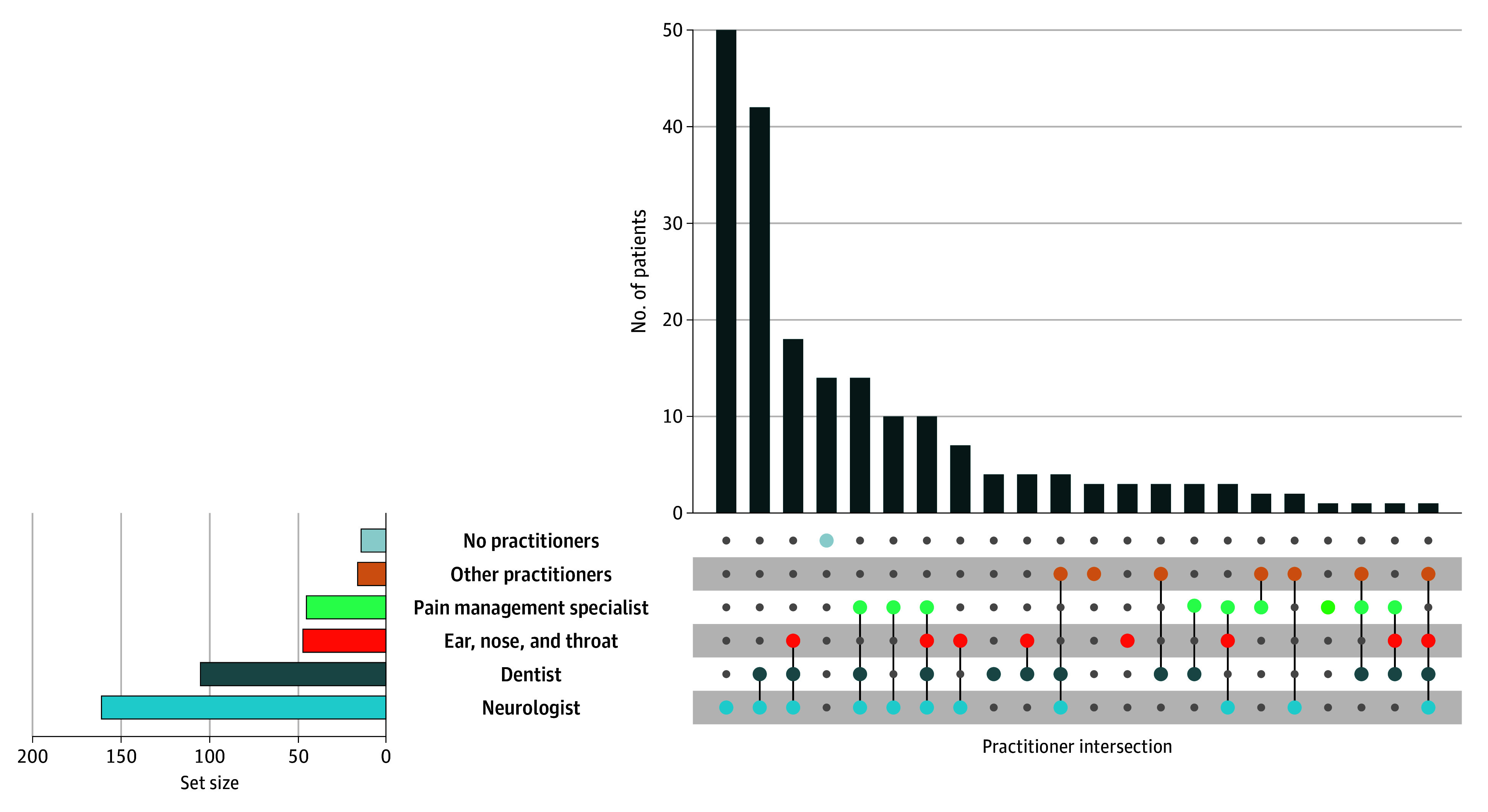
UpSet Plot of Unique Dentist and Medical Practitioner Combinations That Patients With Trigeminal Neuralgia Had Consulted Prior to Neurosurgery The vertical bars of upper right show the number of patients with TN who had previously consulted with the combination of different practitioners noted in each column. The intersection of dots indicate the combination of practitioners that each patient with trigeminal neuralgia in the group had consulted. The set size bar graph depicts the total number of patients with trigeminal neuralgia who had seen each practitioner, independent of other practitioners the patient may have consulted with. Other practitioners include ophthalmologists, chiropractors, temporomandibular joint specialists, radiation oncologists, and physical medicine and rehabilitation.

**Table.** Demographic and Clinical Characteristics of 197 Patients With TN Seeking First Time Neurosurgical Consultation From March 1, 2024, to February 28, 2025^a^

Variable	No. (%)	*P* value
Age, median (IQR), y	62.4 (47.9-69.9)	NA
Sex		
Male	68 (35.9)	NA
Female	127 (64.1)	NA
Distribution of pain		
V1	87 (44.2)	NA
V2	163 (82.7)	NA
V3	130 (66.0)	NA
BNI pain score^b^		
≤IIIa	6 (3.0)	NA
IIIb	7 (3.5)	NA
IV	75 (38.1)	NA
V	108 (54.8)	NA
Patients with prior dental visit (n = 105)		
Prior dental procedures	31 (29.5)	NA
Tooth removal (nonwisdom teeth)	20 (19.0)	NA
Root canal	11 (10.5)	NA
Other dental procedure^c^	10 (9.5)	NA
Multiple	10 (9.5)	NA
Dental referral to neurology/neurosurgery	27 (25.7)	NA
Patients with prior nondental nonneurosurgical visits for TN pain (n = 187)		
No. of specialists seen, median (IQR)	3 (2-4)	NA
Neurologist	161 (86.1)	NA
ENT	47 (25.1)	NA
Pain management	45 (24.1)	NA
Primary care	17 (9.1)	NA
Other^d^	15 (8.0)	NA
Prior nondental nonneurosurgical procedure	52 (27.8)	NA
OnabotulinumtoxinA (Botox)	7 (13.5)	NA
Nerve block	21 (40.4)	NA
Sinus surgery	7 (13.5)	NA
Acupuncture	6 (11.5)	NA
Other^e^	6 (11.5)	NA
Multiple procedures	31 (16.6)	NA
Symptom duration until first neurosurgical consult, median (IQR), mo		
All patients with TN	36 (12-83)	
Consulted a dentist	37 (12-90)	.47
Did not consult a dentist	36 (12-72)	
Had a dental procedure	46.5 (25-119.7)	.29
Did not have a dental procedure	38 (12-82.8)	
Dentist diagnosed TN	45 (6.5-55)	.41
Dentist did not diagnose TN	36 (12-96)	
Had a medical procedure	52 (27-120)	.03
Did not have a medical procedure	36 (11-72)	
Consulted >1 medical specialists	59 (30-119.7)	.01
Consulted 0-1 multiple medical specialists	36 (11.2-80.5)	

Abbreviations: BNI, Barrow Neurological Institute; ENT, ears, nose, and throat; NA, not applicable; TN, trigeminal neuralgia; V1, first branch of the trigeminal nerve; V2, second branch of the trigeminal nerve; V3, third branch of the trigeminal nerve.

^a^ Description of medical practitioner visits prior to first neurosurgical consultation are included for applicable patients in addition to duration of symptoms, in months, from onset of TN pain to the time of first neurosurgical consultation based on different pre-neurosurgical care factors. The number of medical specialists consulted and number of medical procedures are associated with an increased duration of symptoms, meanwhile dental based factors do not significantly delay time to neurosurgical consultation.

^b^ Range, I to V, with I indicating no pain and V severe pain.

^c^ This category includes wisdom teeth removal (3 patients), apicoectomy (1 patient), cavity refill (1 patient), V3 nerve blocks (1 patient), resection of gum bone spurs (1 patient), implants (2 patients), and night guard given (1 patient).

^d^ This category includes emergency medicine; chiropractor; ophthalmologist; temporomandibular joint specialists, and physical medicine and rehabilitation.

^e^ This category includes allergy shots, odontogenic keratocyst resection, intravenous ketamine, and antibiotics.

Most patients (187 [94.9%]) sought other medical practitioners prior to neurosurgical consultation and 52 (27.8%) of them had nondental procedures. The Table summarizes care received prior to neurosurgical consultation. Patients with prior nondental procedures experienced an increased symptom duration (no prior procedure: median, 36 [IQR, 11-72] months; prior procedure: 52 [IQR, 27-120] months; *P* = .03), along with those who saw multiple specialists (0-1 specialist: median, 36 [IQR, 11.2-80.5] months; >1 specialist: 59 [IQR, 30-119.7] months; *P* = .01).

## Discussion

Our findings illustrate the various practitioner pathways patients with TN take prior to neurosurgical consultation. Most of these patients seek dental care, and many undergo dental procedures to alleviate TN facial pain. Limitations include the single-center design and self-reported data, which may introduce recall bias in patient-reported timelines and interventions. Dentists play a vital role in diagnosing and referring patients with TN after ruling out odontogenic causes. Although TN facial pain is traditionally taught as distinct from dental pain, the differences may not be clearly obvious in clinical practice. A cross-sectional survey of 229 dentists in Turkey found that although 82% reported familiarity with TN diagnostic criteria, nearly half (45.9%) confused it with dental pain.^2^ This underscores the need for enhanced TN education across all health care disciplines, especially as prior studies revealed that 60 to 90% of patients consulted dentists and up to 48% underwent dental procedures prior to receiving a TN diagnosis.^3,4,5^

Notably, our results demonstrated that prior dentist visits, procedures, or misdiagnosis by dentists did not significantly delay time to neurosurgical consultation. Beyond dental care, multiple specialist visits and nondental interventions were associated with longer preoperative symptom durations, which have been associated with worse postoperative outcomes for TN.^6^ This highlights a target for intervention that would facilitate earlier recognition and streamlined referral pathways for patients with TN. Incorporating comprehensive TN education into interdisciplinary training and developing clinical tools to aid in differentiating TN from odontogenic or other facial pain syndromes may improve timely diagnosis and care. Future work should focus on identifying which patients benefit most from consultation with specific specialists, including neurosurgery.

## Notes

Supplement.
Data Sharing Statement
