# Supplementary material for: Preneurosurgical Care for Patients With Trigeminal Neuralgia
Source: JAMA Netw Open. 2026 Feb 12;9(2):e2558967. doi: 10.1001/jamanetworkopen.2025.58967 (PMC12902889; doi:10.1001/jamanetworkopen.2025.58967)
Supplement: Supplement. — Data Sharing Statement [file jamanetwopen-e2558967-s001.pdf]

Data Sharing Statement

Alfonzo Horowitz. Preneurosurgical Care for Patients With Trigeminal Neuralgia. *JAMA Netw Open*. Published February 12, 2026. doi:10.1001/jamanetworkopen.2025.58967

Data

Data available: Due to encrypted research database, data cannot be shared publicly because of the policy by HWDC, MoHW based on the Personal Data Protection Act. Contact information for data application: https://dep.mohw.gov.tw/dos/cp-5119-59201-113.html. All databases were encrypted due to privacy concerns but linkable for research purposes and limited to use at the Health and Welfare Data Center (HWDC) only.
